# A Nomogram Predicting the Prognosis of Children With Biliary Atresia After Hepatoportoenterostomy

**DOI:** 10.3389/fped.2021.641318

**Published:** 2021-02-24

**Authors:** Jin-qiao Liu, Wen-juan Chen, Meng-jie Zhou, Wen-feng Li, Ju Tang, Qi-chang Zhou

**Affiliations:** ^1^Department of Ultrasound, The Second Xiangya Hospital of Central South University, Changsha, China; ^2^Department of Ultrasound, Hunan Children's Hospital, Changsha, China

**Keywords:** biliary atresia, kasai portoenterostomy, liver transplantation, prognosis, LSM, nomogram

## Abstract

**Background:** Although Kasai portoenterostomy (KPE) is performed timely for most children with biliary atresia (BA), the native liver survival (NLS) is still poor due to the progressive liver fibrosis. Many children have to receive liver transplantation (LT) within 2 years after KPE. Early prediction of the prognosis permits the implementation of prophylactic treatments for BA children. However, studies about the prediction are limited.

**Objective:** The purpose of this study is to establish a nomogram to predict the prognosis of BA children within 2 years after KPE.

**Methods:** The follow-up data of 151 BA children were retrospectively reviewed, and were randomly divided into a training cohort for constructing a nomogram (*n* = 103) and a validation cohort (*n* = 48). In the training cohort, patients were divided into Group A and Group B according to whether death or LT were observed within 2 years post-KPE. Multivariate Cox regression based on the baseline characteristics, liver function indicators and LSM (liver stiffness measurement) values at KPE and 3 months after KPE was utilized for the establishment of the nomogram in predicting the prognosis of BA within 2 years after KPE. The discrimination and calibration of the nomogram were internally and externally validated.

**Results:** Fifty-six BA children were included in Group A and 47 were included in group B. Age at KPE, METAVIR score F4, LSM at 3 months, first onset of cholangitis within 3 months, and jaundice clearance time were the independent predictors for the prognosis of BA children within 2 years after KPE (all *P* < 0.05). The developed nomogram based on these independent predictors showed good discrimination and calibration by the internal and external validation. Its performance was better than each predictor in predicting the prognosis (all *P* < 0.05).

**Conclusions:** The established nomogram based on the indicators from the first 3 months after KPE may be useful for predicting the prognosis of BA children within 2 years post-KPE and helpful for the consideration of LT.

## Introduction

The pathogenesis of biliary atresia (BA) involves a progressive fibro-inflammatory process affecting intrahepatic and extrahepatic bile ducts. If untreated, BA children eventually progress to cirrhosis before the age of 2 years (1, 2). Kasai portoenterostomy (KPE) is recognized as the initial choice after the diagnosis of BA. However, liver transplantation (LT) is still required on 70% of children despite the timely performance of KPE (3). It is believed that the cause of BA may involve a persistent inflammatory process rather than a simple obliteration of biliary ducts, which may be mediated by autoimmunity (4, 5). Hence, progressive liver fibrosis may still occur in postoperative children, which may lead to poor native liver survival (NLS) and poor long-term prognosis (6). Besides, several risk factors, such as age at KPE (7), serum bilirubin levels (8), cholangitis (9) are predictive for the prognosis of BA children after KPE. Therefore, postoperative monitoring of liver fibrosis in BA children is important. Liver biopsy is not safe for BA children and the obtained histological material is insufficient although it is the gold standard for fibrosis (10). Liver stiffness measurement (LSM), obtained by shear wave elastography (SWE) has become a reliable and popular non-invasive means in assessing hepatic fibrosis, which has been used for the differential diagnosis of BA (11–13). At present, elastography is applied in the post-KPE follow-up in some studies, and their LSM values correlate well with the histological stage of liver fibrosis, indicating good performance in the assessment of postoperative liver fibrosis (14, 15). Chen et al. (16) found that the correlation between fibrosis stage and LSM values was better than laboratory tests, which indicated that LSM was a more promising manner to assess liver fibrosis. However, few studies have focused on the prediction of the post-KPE complications or the prognosis of BA children utilizing elastography to determine the optimal timing of LT. Although Nightingale et al. (17) proposed in a relatively large (>200) multicenter study that albumin <35 g/L in BA children at 3 months post-KPE was a poor prognostic indicator, it was not enough to influence the decision of earlier LT.

We therefore retrospectively analyzed the LSM values, serological and clinical indicators detected within the first 3 months after KPE in an attempt to establish a nomogram to predict the prognosis of BA children within 2 years after KPE.

## Materials and Methods

### Patient Selection

This study was approved by the ethical review committee of our hospital (HCHLL-2020-99) and conducted following the declaration of Helsinki. The medical records of 151 consecutive pediatric patients who were diagnosed with BA and underwent KPE between April 2015 and April 2018 at Hunan children's hospital were reviewed retrospectively. Complete follow-up of each BA child was available for at least 2 years. The exclusion criteria were as follows: (1) patients who were unable to survive with native liver within 3 months post-KPE; (2) patients who were coinfected with hepatitis B or hepatitis C; and (3) patients who had a history of hepatic decompensation. The eligible patients were randomly divided into a training cohort for constructing a nomogram (*n* = 103) and a validation cohort (*n* = 48). The baseline characteristics including age at KPE, gender, BA classification (I–III) were recorded. Hepatic fibrosis was assessed histologically by wedge biopsy during the KPE and graded using the METAVIR score (F0-F4) (18).

### Assessment of SWE

**Figure 1:**
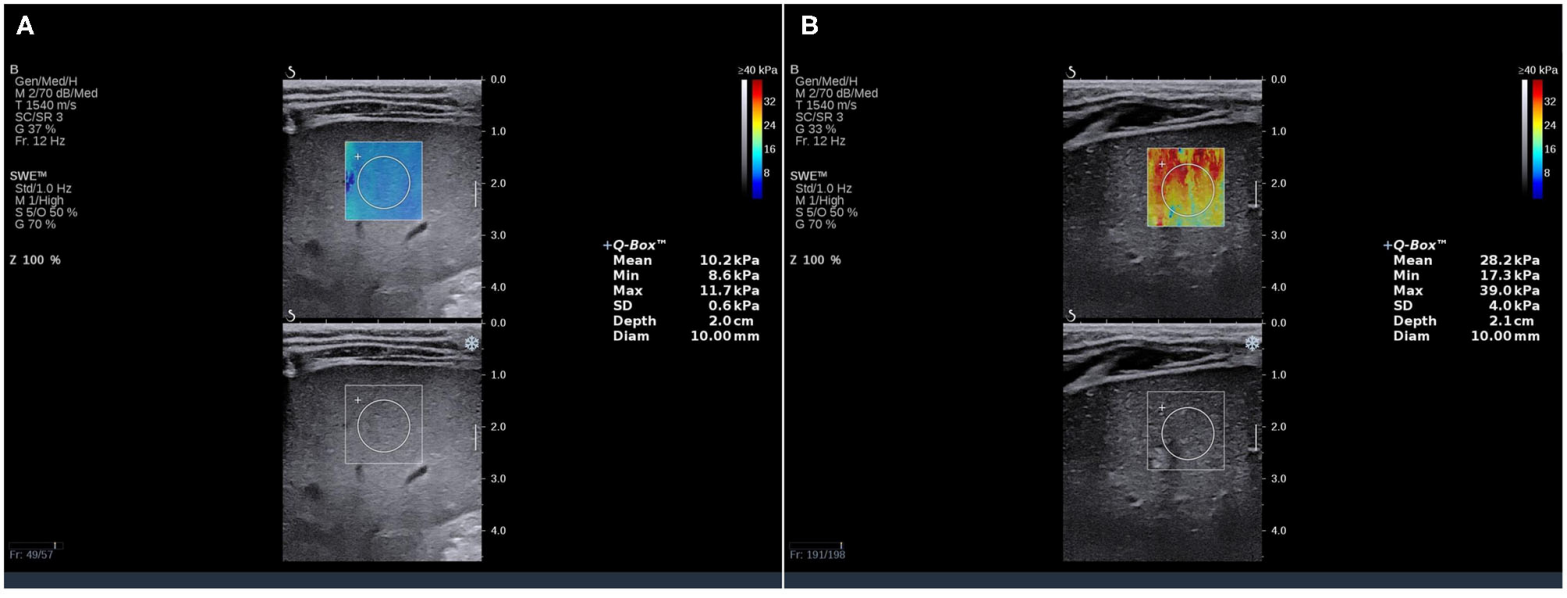
SWE measurements in BA children. The SWE showed a stiffness color map (top) and a grayscale image (bottom). The LSM value in the ROI of **(A)** was 10.2 kPa (METAVIR score F2), and **(B)** was 28.2 kPa (METAVIR score F4). SWE, shear wave elatography; LSM, liver stiffness measurement; ROI, region of interest.

LSM values were obtained using Aixplorer ultrasound system (SuperSonic Imagine, Aix-en-Provence, France) after routine abdominal examinations. The measurements were taken by two sonographers with more than 10 years of experience in abdominal ultrasound and more than 3 years of experience in SWE who were blinded to the METAVIR scores. LSM was measured through the intercostal spaces when the child lay in the supine position with peaceful breathing. The measurement depth was between 2.5 and 5.5 cm below the skin surface. The range of the SWE sampling frame (2 × 2 cm) was set 1 cm below the liver capsule of the right anterior lobe. Vessels, intrahepatic biliary tracts, and gallbladder were avoided. A circular region of interest (ROI) (10 mm in diameter) was positioned in the sampling frame. A valid SWE was achieved when most of the ROI (>90%) was filled with homogeneous color. Five valid measurements were recorded for each child within 5 min, and the median value was considered representative of the elastic modulus of the liver and expressed in kilopascals (kPa) (Figure 1).

### Liver Function Test

Liver function tests were performed at KPE and during the follow-up. Data including aspartate aminotransferase (AST), alanine aminotransferase (ALT), albumin (ALB), total bilirubin (TBil), direct bilirubin (DBil), gamma-glutamyl transpeptidase (GGT), alkaline phosphatase (ALP), prothrombin time (PT-INR), and platelet count (PLT) were recorded.

### Follow-Up and Patient Grouping

All pediatric patients received a unified schedule for anti-inflammatory, choleretic, and hepatoprotective treatments after receiving KPE. Regular follow-ups were performed for all BA children in our institution. In the training cohort, death or LT in BA children within 2 years were included in Group A. Those without any liver-related event were included in Group B. LT was indicated if met at least one of the following criteria: worsening portal hypertension, refractory ascites, upper gastrointestinal bleeding, repetitive bouts of cholangitis, or liver failure. Liver function indicators and LSM values were recorded and compared before KPE, at KPE, 2 weeks, 1 month, 2 months, and 3 months after KPE.

### Statistical Analysis

The numerical data were expressed as mean ± standard deviations for normally distributed variables and as median (interquartile range) for non-normally distributed variables. Independent sample *t*-test and Mann-Whitney *U*-test were used for comparison. The categorical variables were expressed as number (percentage) and the chi-square test was used for comparison. The cumulative NLS was calculated using the Kaplan-Meier (KM) method. The clinical characteristics, liver function indicators, and LSM values were compared between the two groups. The variables that were significant at *P* < 0.05 in the comparison included in the multivariate Cox proportional hazards regression model.

A nomogram was developed based on the weighted sum of each independent variable, with the weights equal to the hazard ratios from the multivariate Cox model to predict the prognosis of BA children within 2 years after KPE. The nomogram was internally validated in the training cohort and externally validated in the validation cohort. Bootstrap resampling (1,000 times) analysis was utilized to obtain relatively unbiased estimates of the performance of the model. The discrimination of the nomogram was evaluated by the area under the curve (AUC) of receiver operating characteristic (ROC) curve (AUC > 0.75 indicating good discrimination). A calibration curve, which plotted the average Kaplan–Meier estimate against the corresponding prognosis predicted by the nomogram, combined with the Hosmer–Lemeshow (HL) test were utilized to evaluate the calibration of the nomogram (19). IBM SPSS Statistics 22.0 (IBM Corp., Armonk, NY, United States), R package version 3.6.2. and Medcalc (Version 22.0.1; MedCalc Software, Ostend, Belgium) were used for statistical analyses.

## Results

### Characteristics and NLS Within 2 Years After KPE in the Training and Validation Cohorts

**Figure 2:**
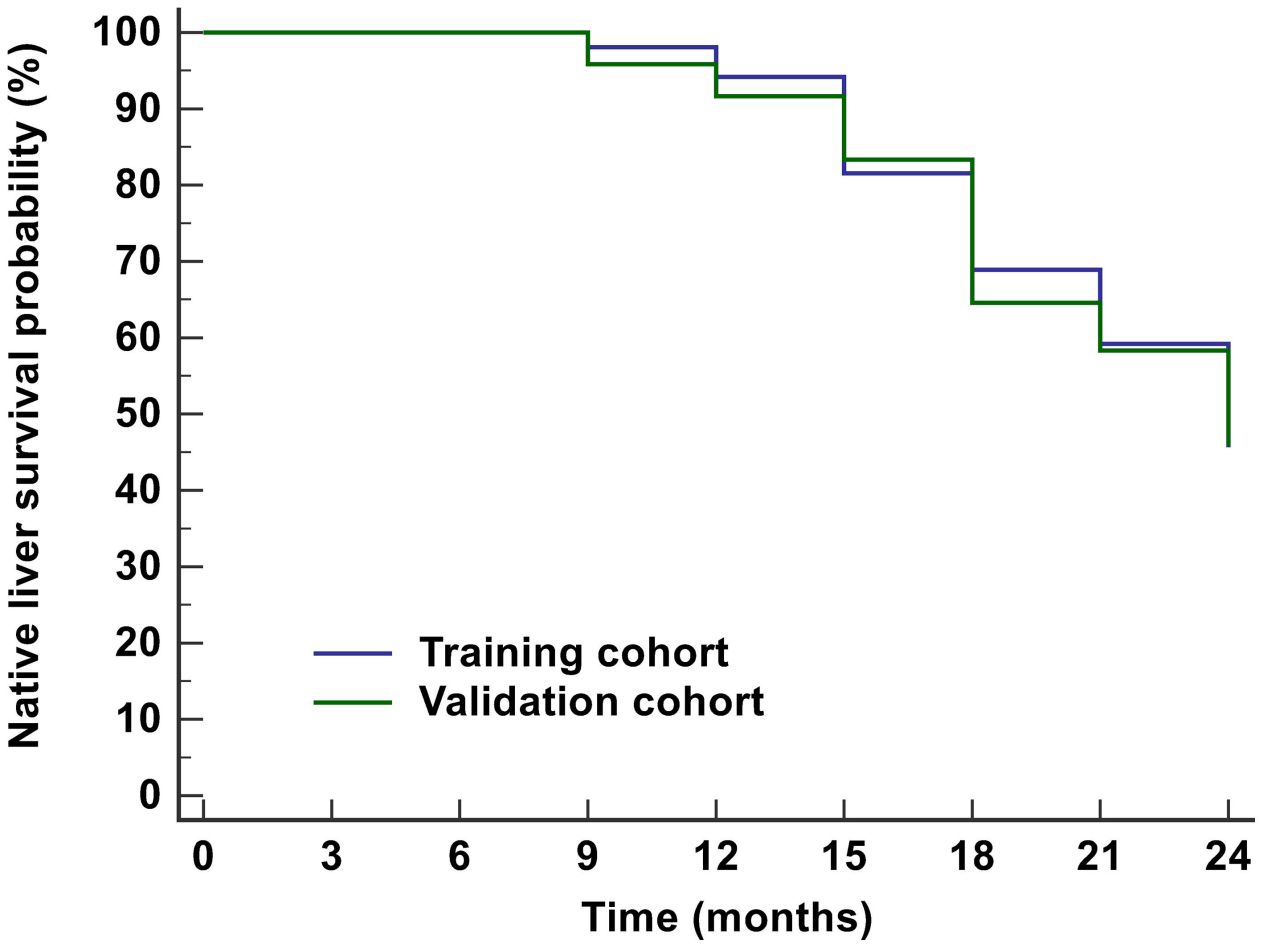
Kaplan-Meier analysis for the prognosis of BA children within 2 years after KPE. The NLS probabilities in the training cohort and validation cohort were 45.6 and 45.8%, respectively. BA, biliary atresia; KPE, Kasai portoenterostomy; NLS, native liver survival.

The characteristics of the training and validation datasets were comparable in both cohorts, justifying their use as training and validation datasets. There was no significant difference in NLS within 2 years between the training (45.6%) and validation datasets (45.8%) (Figure 2).

### Baseline Characteristics of the Training Cohort Between the Two Groups

Of the 103 BA children in the training cohort (47 male and 56 female, mean age 65.21 ± 20.14 days), 56 were included in Group A and 47 were in Group B. The comparison of baseline characteristics between the two groups are shown in Table 1. The age at KPE and the proportion of METAVIR score F3 and F4 in Group A were higher than those in Group B (*P* < 0.05).

**Table 1:** Comparison of baseline characteristics between Group A and Group B.

**Characteristics**		**Group A (*n* = 56)**	**Group B (*n* = 47)**	***P***
Age at KPE (days)		72.02 ± 19.47	59.38 ± 20.46	0.002^*^
Gender	Male	23 (41.1%)	24 (51.1%)	0.311^$^
	Female	33 (58.9%)	23 (48.9%)	
METAVIR score	F0	0 (0%)	0 (0%)	0.015^#^
	F1	0 (0%)	0 (0%)	
	F2	5 (8.9%)	11 (23.4%)	
	F3	21 (37.5%)	23 (48.9%)	
	F4	30 (53.6%)	13 (27.7%)	
BA classification	I	0 (0%)	0 (0%)	0.858^#^
	II	2 (3.6%)	2 (4.3%)	
	III	54 (96.4%)	45 (95.7%)	

*BA, biliary atresia; KPE, Kasai portoenterostomy; Group A, BA children underwent LT or died within 2 years; Group B, BA children alive with their native livers within 2 years*.

* *for independent sample t-test*,

$ *for chi-square test*,

# *for Mann-Whitney U-test*.

### Trends of LSM, ALT, and TBil Within 3 Months of Follow-Up

**Figure 3:**
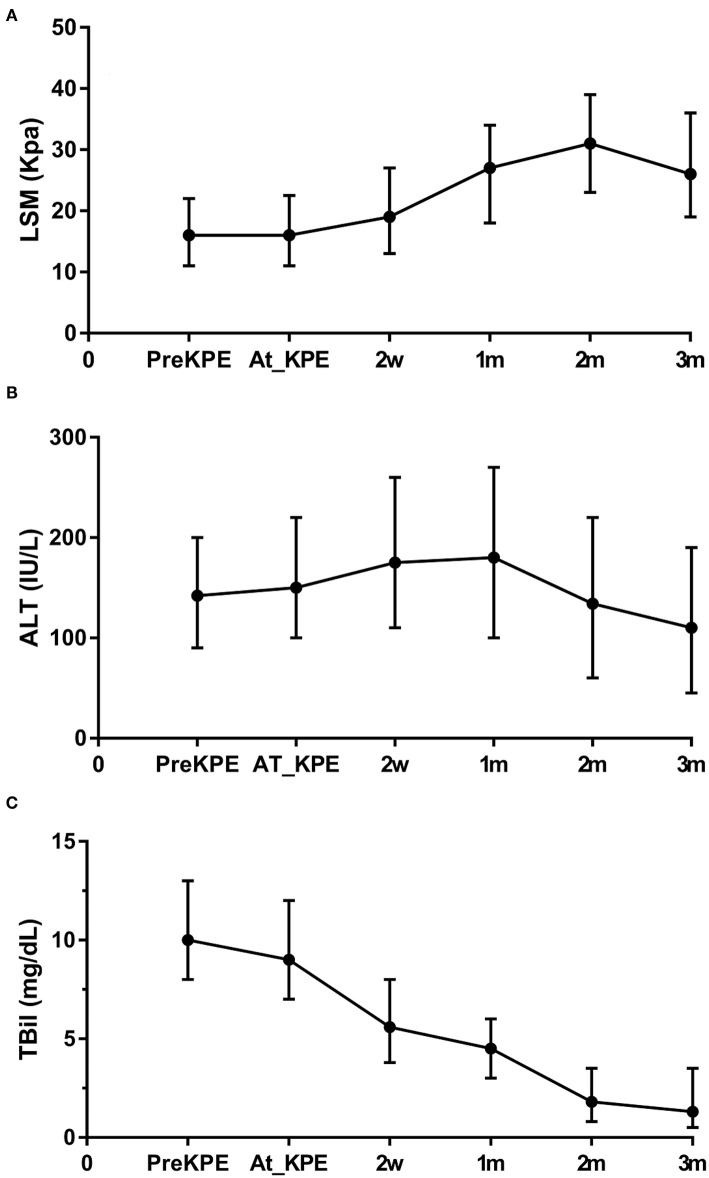
Trends of LSM, ALT, and TBil within 3 months of follow-up post-KPE. The general trends of LSM **(A)** and ALT **(B)** were rising first and then decreasing. TBil **(C)** showed a significant decreasing trend after KPE. However, the 75th percentiles of TBil at 2 and 3 months after KPE increased. LSM, liver stiffness measurement; ALT, aspartate aminotransferase; TBil, total billrubin.

The trends of LSM, ALT, and TBil within 3 months of follow-up are shown in Figure 3. The general trends of LSM and ALT were rising first and then decreasing. The peak values of ALT and LSM appeared at 1 and 2 months, respectively. The mean values of ALT and LSM within 3 months were greater than the upper limit of normal (ALT: 40 UI/L, LSM: 5 kPa). TBil showed a significant decreasing trend after KPE, and the average value remained within the normal range after 2 months (<2 mg/dL). However, the 75th percentiles of TBil at 2 and 3 months after KPE gradually increased.

### Comparison of the Two Groups at Time of KPE and 3 Months After KPE

At the time of KPE, the LSM value in Group A was higher than Group B (*P* < 0.05). When 3 months after KPE, the LSM value, AST, TBil, DBil, the proportion of first onset of cholangitis, and jaundice clearance time were greater than those in Group B (all *P* < 0.05) (Table 2).

**Table 2:** Comparison of the two groups at time of KPE and 3 months after KPE.

**Variables**	**At the time of KPE**			**3 months after KPE**		
	**Group A (*n* = 56)**	**Group B (*n* = 47)**	***P***	**Group A (*n* = 56)**	**Group B (*n* = 47)**	***P***
LSM value (kPa)	15.42 ± 4.13	13.21 ± 5.15	0.017^*^	28.25 ± 8.12	23.50 ± 7.04	0.024^*^
AST (IU/L)	171.70 ± 48.75	160.60 ± 53.10	0.272^*^	161.37 ± 50.18	113.19 ± 37.25	0.043^*^
ALT (IU/L)	166.13 ± 56.3	151.34 ± 57.31	0.190^*^	137.83 ± 55.11	87.31 ± 41.17	0.017^*^
Albumin (g/dL)	3.22 ± 0.46	3.47 ± 0.53	0.162^*^	3.43 ± 0.45	3.74 ± 0.78	0.084^*^
TBil (mg/dL)	9.88 ± 3.55	8.96 ± 2.84	0.156^*^	1.48 ± 0.49	0.98 ± 0.27	0.005^*^
DBil (mg/dL)	8.18 ± 2.93	7.45 ± 2.43	0.178^*^	1.05 ± 0.40	0.84 ± 0.23	0.008^*^
GGT (IU/L)	516.32 ± 142.76	530.97 ± 123.80	0.583^*^	489.50 ± 137.02	451.08 ± 116.98	0.133^*^
ALP (IU/L)	625.12 ± 173.48	577.53 ± 192.76	0.190^*^	525.96 ± 155.27	483.46 ± 122.30	0.120^*^
PT-INR	1.001 ± 0.197	0.983 ± 0.221	0.661^*^	1.145 ± 0.199	0.982 ± 0.138	0.215^*^
PLT(10^9^/L)	249.78 ± 82.29	263.66 ± 69.74	0.357^*^	253.44 ± 75.82	266.32 ± 95.23	0.456^*^
Cholangitis [n (%)]	–	–	–	19 (33.9%)	6 (12.8%)	0.013^$^
Multiple cholangitis episodes (≥2) [n (%)]				3 (5.36%)	0 (0%)	0.109^#^
Jaundice clearance time (days)	–	–	–	33 (21, 48)	20 (14, 25)	<0.001^$^

*BA, biliary atresia; KPE, Kasai portoenterostomy; LSM, liver stiffness measurement; AST, aspartate aminotransferase; ALT, alanine amino-transferase; TBil, total billrubin; DBil, direct billrubin; GGT, gamma-glutamyl transpeptidase; ALP, alkaline phosphatase; PT-INR, prothrombin time; PLT, platelet count; Group A, BA children underwent LT or died without LT within 2 years; Group B, BA children alive with their native livers within 2 years*.

* *for independent sample t-test*,

$ *for chi-square test*,

# *for Mann-Whitney U-test*.

### Independent Predictors Associated With the Prognosis Within 2 Year Post-KPE

**Figure 4:**
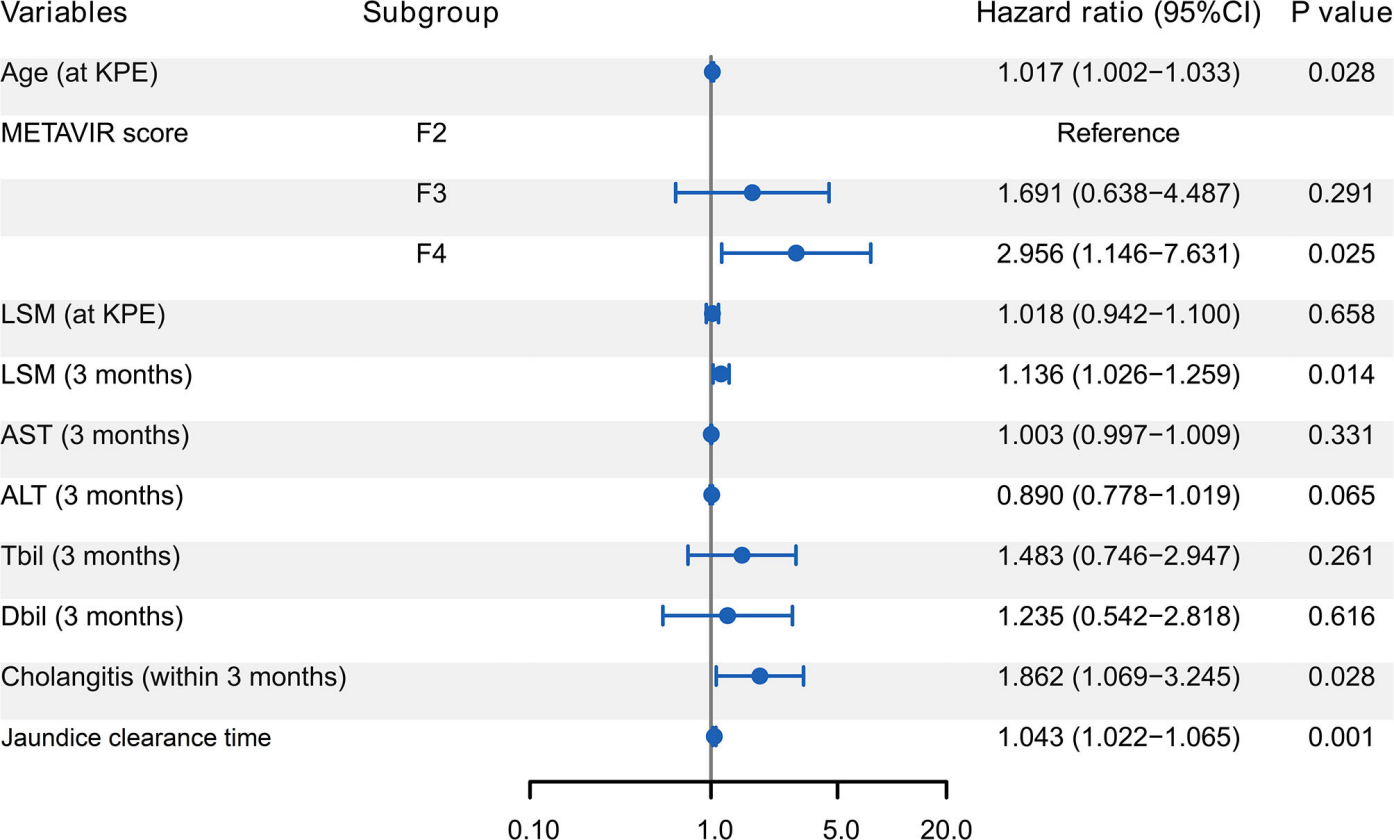
Forest plot of Cox regression analysis for predictors of the prognosis of BA children within 2 year post-KPE. Age at KPE, METAVIR score F4, LSM at 3 months, first onset of cholangitis within 3 months, and jaundice clearance time were the independent predictors. BA, biliary atresia; KPE, Kasai portoenterostomy; LSM, liver stiffness measurement; AST, aspartate aminotransferase; ALT, alanine amino-transferase; TBil, total billrubin; DBil, direct billrubin.

Multivariate Cox regression further revealed that age at KPE, METAVIR score F4, LSM at 3 months, first onset of cholangitis within 3 months, and jaundice clearance time were the independent predictors for the prognosis of BA children within 2 years after KPE (Figure 4).

### Nomogram Development

**Figure 5:**
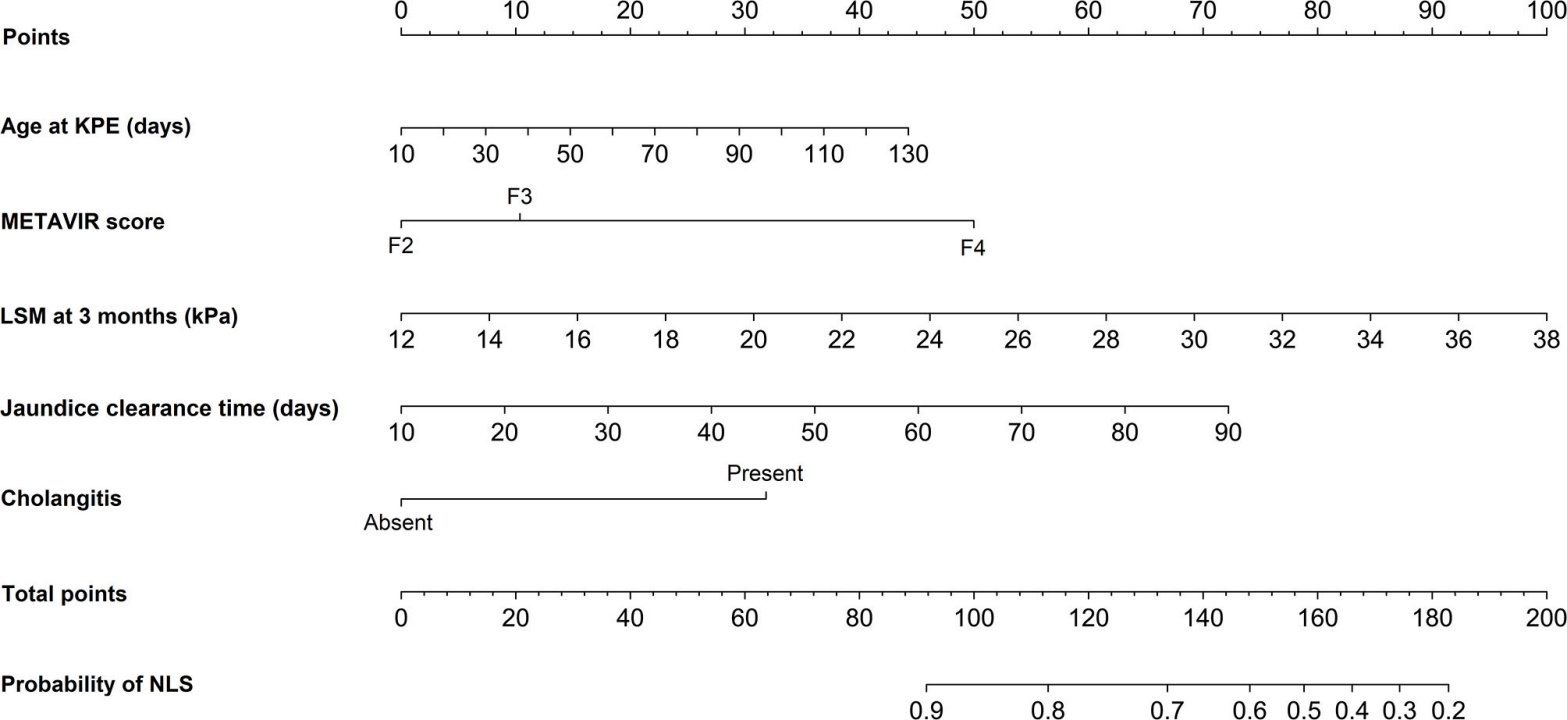
Nomogram for predicting the probability of NLS in BA children within 2 years post-KPE. The top row showed the point assignment for each variable. Rows 2–6 indicated the variables included in the nomogram. The bottom row showed the probability of NLS. BA, biliary atresia; KPE, Kasai portoenterostomy; NLS, native liver survival; LSM, liver stiffness measurement.

A nomogram incorporating these independent predictors from the first 3 months after KPE was constructed to predict the probability of NLS within 2 years (Figure 5). Each predictor was assigned a number with the weights equal to the hazard ratios from the multivariate Cox model and the estimated probability of NLS in a BA child was determined by adding the scores of each predictor. For example, a BA child with a METAVIR score F3 (10 points) received KPE at the age of 60 days (18 points). His jaundice clearance time was 40 days (27 points), and he suffered cholangitis within 3 months (32 points). His LSM value recorded at 3 months post-KPE was 24 kPa (46 points). In that case, the total score was about 133, which indicated that the probability of NLS within 2 years was about 70%.

### Internal and External Validation of the Nomogram

**Figure 6:**
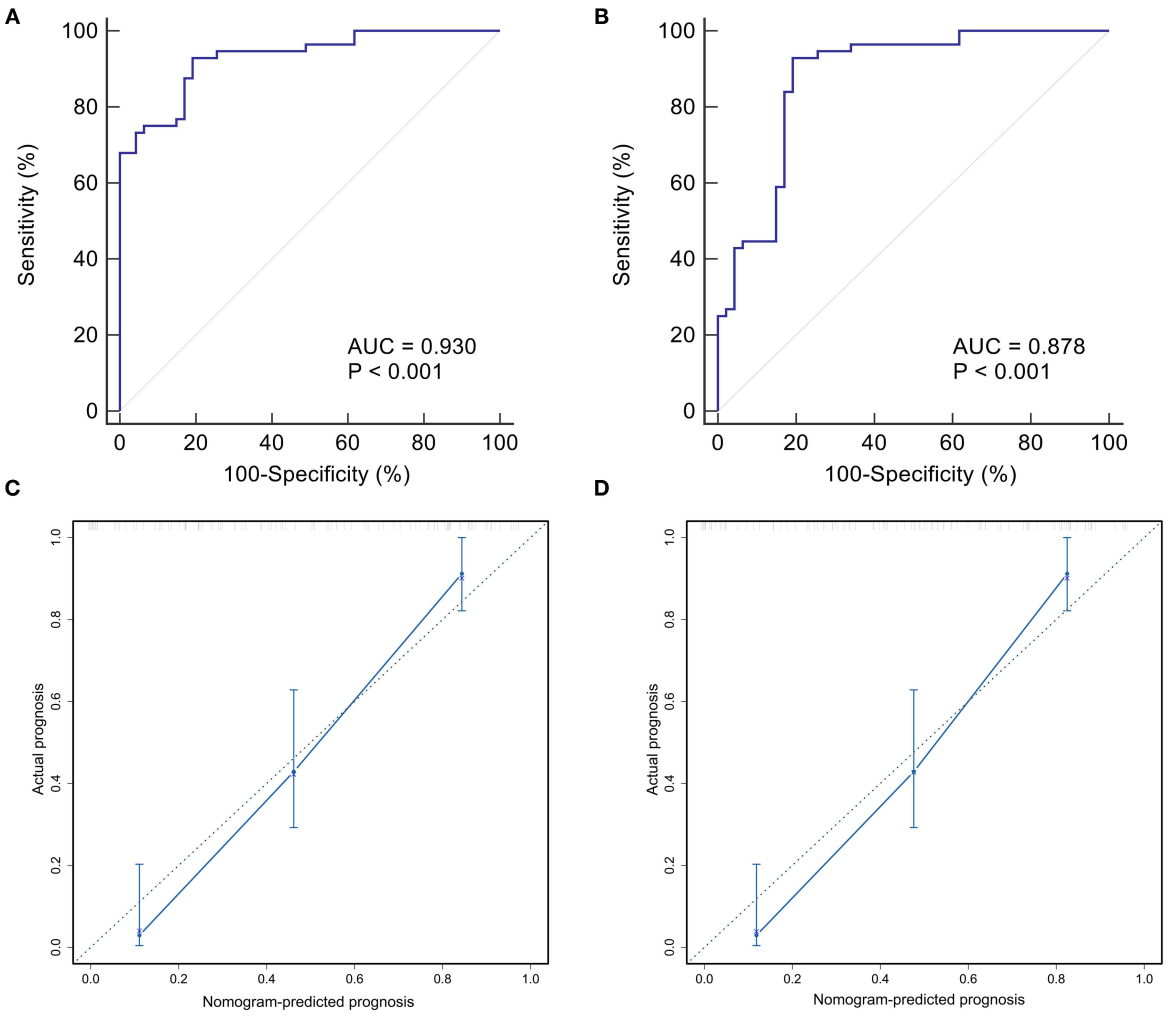
Internal and external validation of the nomogram predicting the prognosis of BA within 2 years post-KPE. The ROC curves were plotted for the discrimination of the nomogram. The AUCs in the training cohort and validation cohort were 0.930 **(A)** and 0.878 **(B)**, respectively, indicting good discrimination. The calibration curves were plotted for evaluating the calibration of nomogram-predicted prognosis and actual prognosis estimated using KM analysis. The calibration plots in the training and validation datasets were shown in **(C,D)**. Both indicated that the nomogram-predicted progonisis compared very well with the actual prognosis. KPE, Kasai portoenterostomy; ROC, receiver operating characteristic curve; AUC, area under the curve; KM, Kaplan-Meier.

In the internal validation, the ROC curve of the nomogram showed quite good discrimination based on the training datasets (AUC = 0.930, Figure 6A). The calibration plot graphically showed that the nomogram-predicted prognosis and actual prognosis estimated using KM analysis agreed well in the training cohort (Figure 6B). The HL test yielded no significant difference between the predicted and actual prognosis (*P* = 0.428), suggesting good fitting of the nomogram. In the external validation using the validation datasets, the nomogram also displayed good discrimination with an AUC of 0.80 (Figure 6C). Good calibration was also demonstrated by a non-statistical significance obtained in the HL test (*P* = 0.356), as displayed by the calibration plot (Figure 6D).

**Figure 7:**
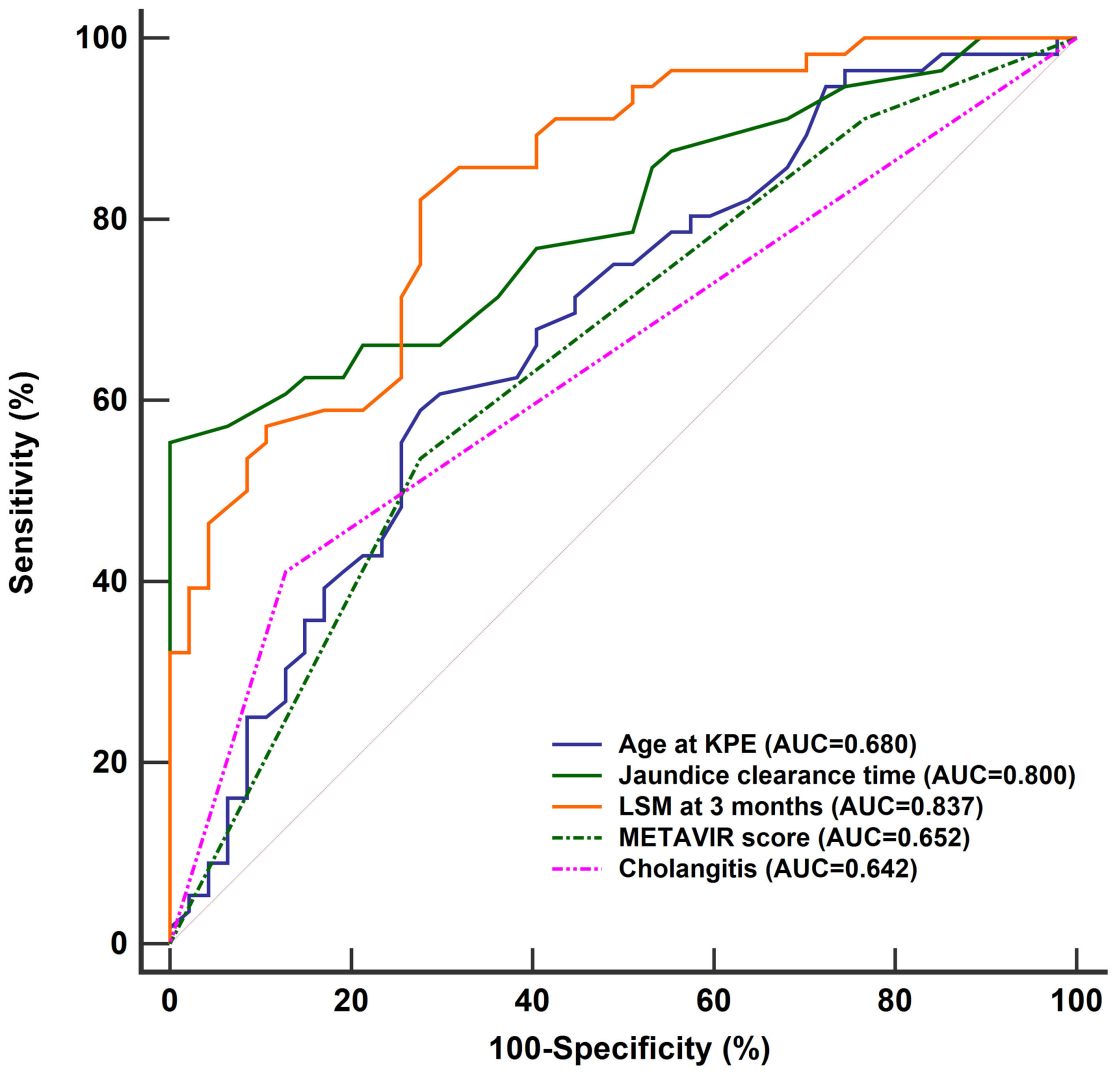
ROC curves of age of KPE, jaundice clearance time, LSM at 3 months, METAVIR score, and first onset of cholangitis within 3 months in predicting the prognosis of BA children within 2 years post-KPE. The AUCs of them [age of KPE: 0.680 (95% CI: 0.581–0.769), jaundice clearance time: 0.800 (95% CI: 0.710–0.872), LSM at 3 months: 0.837 (95% CI: 0.752–0.903), METAVIR score: 0.652 (95% CI: 0.551–0.743), and first onset of cholangitis within 3 months: 0.642, (95% CI: 0.541–0.734)] were lower than the nomogram (all *P* < 0.05). ROC, receiver operating characteristic curve; AUC, area under the curve; KPE, Kasai portoenterostomy; LSM, liver stiffness measurement; CI, confidence interval.

To compare the performance of the nomogram, the accuracies of the independent predictors were analyzed by ROC curves (Figure 7). It showed that the accuracies of independent predictors were lower than the nomogram (all *P* < 0.05), which indicated that each predictor could not accurately predict the prognosis of BA within 2 years post-KPE.

## Discussion

Accurate and early prediction of poor prognosis after KPE permits the implementation of prophylactic treatments for potential complications of progressive liver disease and early consideration of LT (20). In this study, with serial liver function tests and SWE assessments, poor prognosis of BA children within 2 years after KPE was associated with the features such as older age at KPE, METAVIR score F4, longer jaundice clearance time, first onset of cholangitis within 3 months, and higher LSM at 3 months. The developed nomogram based on the indicators within the first 3 months after KPE showed better performance than individual indicators for predicting the prognosis.

Two years post-KPE is a critical period that affects the survival of BA children. The condition of some children will change greatly, leading to a rapid decline in the NLS rate during this period (21). This may be due to the younger age, high incidence of postoperative complications, and loss of confidence in their parents. After 2 years, the decrease in NLS is gradually stabilized and more children are alive with their native livers (8).

Although it is a retrospective study, it is unique because the liver function and LSM within 3 months after KPE were analyzed for predicting the prognosis. As indicated by the general trends of ALT and LSM, hepatic inflammation, cholestasis, and liver fibrosis required resolution within 3 months post-KPE (22). Hence, the mean values of ALT and LSM were higher than the normal healthy children, which was consistent with the report of Tokuhara et al. (23). However, due to the diffuse disease of small intrahepatic bile ducts in some BA children, cholestasis is difficult to improve although the local biliary drainage is unobstructed in the early postoperative period. The worsening process of liver function and liver fibrosis will continue (24). The increased 75th percentiles of TBil at 2 and 3 months after KPE in the current study suggested that some BA children with poor prognosis might be still affected by persistent cholestasis since generally the resolution time of the jaundice after KPE was about 2 months (25). Hence, close monitoring of hepatic disease progression within 3 months after KPE may be valuable for the identification of children with poor outcomes.

Our study revealed that BA children with older age and higher METAVIR score at KPE, longer jaundice clearance time after KPE, and earlier cholangitis were more likely to get a poor postoperative outcomes. However, ROC analyses indicated that their accuracies in predicting the poor prognosis were limited. In addition to these recognized predictors, LSM at 3 months is proved to be predictive for the prognosis of BA as well. Since the introduction of LSM, it has been utilized to monitor the disease progression or predict the onset of progressive liver-related complications of BA post-KPE (26). BA children with higher LSM values are prone to suffer esophageal or gastric varices, leading to poor prognosis in the long-term follow-up (27, 28). However, according to the ROC analysis, LSM at 3 months was not powerful in predicting the prognosis within 2 years post-KPE. This may be because, in addition to the degree of liver fibrosis, LSM is also affected by factors such as inflammation, hepatocyte swelling, and tissue edema (29). Since the liver has just been wounded by KPE, the increase in LSM is often affected by edema and inflammation, which was indicated by the similar trends of ALT and LSM within 3 months. In addition, the accuracy of LSM in evaluating liver fibrosis may be affected by TBil as well. The possible mechanism contributing to the increased LSM is that increased hydrostatic pressure in the biliary system interferes with the conduction of ultrasound in the liver (30). Therefore, although LSM helps predict long-term prognosis, LSM at 3 months is not suitable as an accurate predictor for the prognosis of BA within 2 years.

Until now, few prediction models are developed for the prognosis of BA children within 2 years based on the indicators within 3 months after KPE. The advantage of our nomogram is that all the predictors are convenient to obtain from regular examinations (elastography is now equipped in most ultrasound instruments). The constructed nomogram in the study could be utilized to calculate the scores corresponding to each independent predictor, and the predicted probability corresponding to the sum of the scores was the risk of the poor prognosis. Our study was verified internally and externally that the nomogram had good discrimination and calibration, which allowed a wise decision on the prophylactic treatments for potential complications of progressive liver disease.

Some limitations are inevitable due to the retrospective single-center study. The study population was limited and the selection bias might present since BA children who were not eligible for the study were excluded at first (e.g., severely ill patients). Besides, the interobserver variabilities of LSM were not assessed. A further prospective investigation will help to validate our results on a larger scale to improve the performance of the constructed nomogram.

In conclusion, the established nomogram based on the indicators from the first 3 months after KPE may be used to identify BA children at the risk of poor prognosis within 2 years post-KPE and guide the prophylactic treatments. We believe the liver function and LSM collected within 3 months after KPE may be useful for timely decision making.

## Data Availability Statement

The raw data supporting the conclusions of this article will be made available by the authors, without undue reservation.

## Ethics Statement

The studies involving human participants were reviewed and approved by Ethical approval for the study was obtained from the ethics committee of Hunan Children's Hospital (HCHLL-2020-99). The patients/participants provided their written informed consent to participate in this study.

## Author Contributions

J-qL and Q-cZ: study design, supervision, and manuscript revision. W-jC, M-jZ, W-fL, and JT: data collection and analysis. J-qL, W-jC, M-jZ, W-fL, and JT: statistics. J-qL, W-jC, M-jZ, W-fL, JT, and Q-cZ: manuscript writing. All authors: approval of the manuscript.

## Conflict of Interest

The authors declare that the research was conducted in the absence of any commercial or financial relationships that could be construed as a potential conflict of interest.
